# Decoding the metastatic potential and optimal postoperative adjuvant therapy of melanoma based on metastasis score

**DOI:** 10.1038/s41420-023-01678-6

**Published:** 2023-10-25

**Authors:** Kangjie Shen, Wenyu Song, Hongye Wang, Lu Wang, Yang Yang, Qianrong Hu, Min Ren, Zixu Gao, Qiangcheng Wang, Shaoluan Zheng, Ming Zhu, Yanwen Yang, Yong Zhang, Chuanyuan Wei, Jianying Gu

**Affiliations:** 1 Department of Plastic Surgery, Zhongshan Hospital, Fudan University, Shanghai, China; 2 Department of Cardiovascular Surgery, Zhongshan Hospital, Fudan University, Shanghai, China; 3 Department of Interventional Oncology, Renji Hospital, School of Medicine, Shanghai Jiaotong University, Shanghai, China; 4 The Affiliated Cancer Hospital of Nanjing Medical University, Jiangsu Cancer Hospital, Jiangsu Institute of Cancer Research, Nanjing, China; 5 Department of Plastic and Reconstructive Surgery, Zhongshan Hospital (Xiamen), Fudan University, Xiamen, China; 6 Xiamen Clinical Research Center for Cancer Therapy, Xiamen, China

**Keywords:** Metastasis, Tumour biomarkers

## Abstract

Metastasis is a formidable challenge in the prognosis of melanoma. Accurately predicting the metastatic potential of non-metastatic melanoma (NMM) and determining effective postoperative adjuvant treatments for inhibiting metastasis remain uncertain. In this study, we conducted comprehensive analyses of melanoma metastases using bulk and single-cell RNA sequencing data, enabling the construction of a metastasis score (MET score) through diverse machine-learning algorithms. The reliability and robustness of the MET score were validated using various in vitro assays and in vivo models. Our findings revealed a distinct molecular landscape in metastatic melanoma characterized by the enrichment of metastasis-related pathways, intricate cell–cell communication, and heightened infiltration of pro-angiogenic tumor-associated macrophages compared to NMM. Importantly, patients in the high MET score group exhibited poorer prognoses and an immunosuppressive microenvironment, featuring increased infiltration of regulatory T cells and decreased infiltration of CD8^+^ T cells, compared to the low MET score patient group. Expression of PD-1 was markedly higher in patients with low MET scores. Anti-PD-1 (aPD-1) therapy profoundly affected antitumor immunity activation and metastasis inhibition in these patients. In summary, our study demonstrates the effectiveness of the MET score in predicting melanoma metastatic potential. For patients with low MET scores, aPD-1 therapy may be a potential treatment strategy to inhibit metastasis. Patients with high MET scores may benefit from combination therapies.

## Introduction

Melanoma is projected to cause an estimated 97,610 new cases and 7990 deaths in the United States in 2023 [1]. It is the deadliest of the identified cutaneous malignancies. Following surgery, the prognosis for non-metastatic melanoma (NMM) remains favorable, with a 5-year overall survival (OS) rate of up to 95% [2]. However, the development of tumor metastasis significantly impacts the prognosis for melanoma. Despite the availability of sophisticated molecularly targeted drugs, such as dabrafenib and trametinib, the 5-year OS of metastatic melanoma (MM) is only 34% [3]. Therefore, predicting the metastatic potential of NMM and implementing appropriate postoperative adjuvant treatments to reduce the likelihood of metastasis are crucial scientific challenges to improving patient prognosis.

The utilization of immunotherapy, including immune checkpoint inhibitors (ICIs), tumor-infiltrating lymphocytes, and chimeric antigen receptor T cells, highlights the shift towards precision medicine in treating melanoma [4–6]. In the CheckMate 067 clinical trial, patients with untreated and unresectable MM who received a combination therapy with nivolumab and ipilimumab displayed a 6.5-year OS of 57% in those with *BRAF*-mutant and 46% in those with *BRAF*-wild-type tumors [7]. However, it remains unclear whether immunotherapy could be used as postoperative adjuvant treatment for NMM to inhibit metastasis with any potential benefits for patients [8, 9].

Previous studies primarily focused on decoding melanoma metastasis from a single molecule or single pathway perspective [10–12]. However, the tumor microenvironment (TME) of melanoma is complex and dynamic, with intricate crosstalk between cells, resulting in significant losses of key metastasis-related information. The emergence of high-throughput sequencing enabled the discovery of the evolutionary mechanisms of melanoma metastasis in a new dimension. Bulk RNA sequencing (bulk-seq) has enabled the profiling of gene expression and large-scale clinical cohorts; however, bulk-seq masks intercellular heterogeneity [13, 14]. Single-cell RNA sequencing (scRNA-seq) enables the characterization of the transcriptional profiles of individual cells [15, 16]. Combining bulk- and scRNA-seq enables the appropriate use of clinical features from large cohorts and transcriptional data from individual cells.

In the present study, we utilized both bulk- and scRNA-seq data of cross-species to construct the metastasis score (MET score). The MET score could predict the OS, metastatic potential, and response rate to anti-PD-1 (aPD-1) in melanoma patients. We further validated the accuracy of the MET score using various in vitro and in vivo assays. Our findings suggest that using aPD-1 as a postoperative adjuvant treatment can effectively inhibit melanoma metastasis in patients with a low MET score. Moreover, combination therapy is recommended for patients with a high MET score.

## Results

### Bulk-seq reveals a distinct but contradictory molecular landscape of NMM and MM

**Fig. 1:**
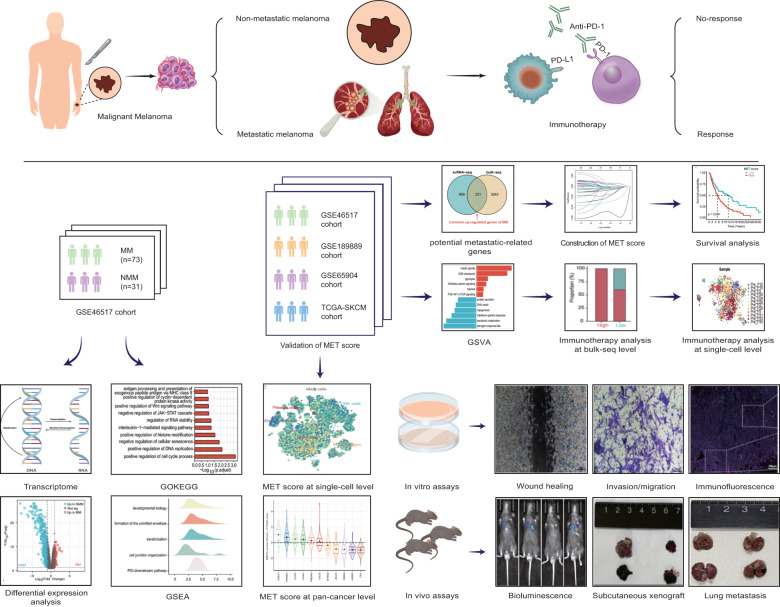
Schematic diagram of the workflow of the study. The metastasis score was constructed using bulk and single-cell RNA sequencing data through diverse machine-learning algorithms and verified by various in vitro assays and in vivo models.

**Fig. 2:**
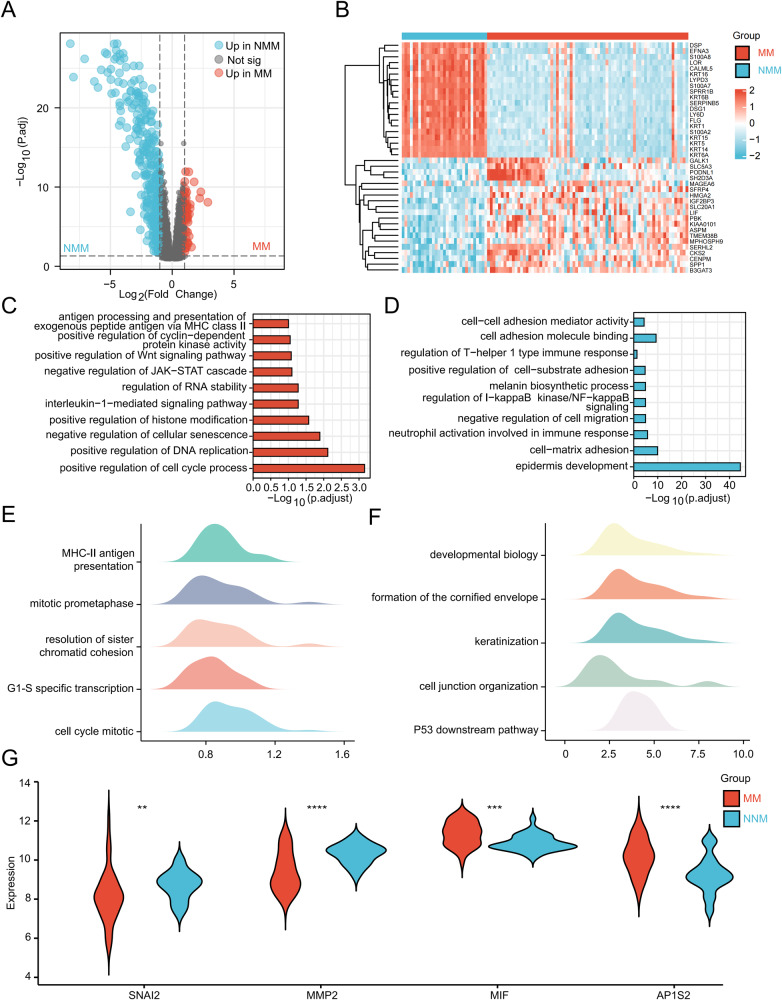
Distinct but contradictory molecular patterns of NMM and MM at bulk-seq resolution. **A**, **B** Volcano plot (**A**) and heatmap (**B**) showing the up-regulated genes in NMM and MM. **C**, **D** GO and KEGG analyses depicting the enriched pathways and biological function in MM (**C**) and NMM (**D**). **E**, **F** GSEA showing the enriched gene lists in MM (**E**) and NMM (**F**). **G** Different expression levels of four metastasis-related genes between MM and NMM. NMM non-metastatic melanoma, MM metastatic melanoma, bulk-seq bulk RNA sequencing, GO Gene Ontology, KEGG Kyoto Encyclopedia of Genes and Genomes, GSEA gene set enrichment analysis. *P < 0.05, **P < 0.01, ***P < 0.001, ****P < 0.0001.

To decode the metastasis of melanoma, integrated analysis of cross-species bulk- and scRNA-seq data, in vitro and in vivo assays were performed. The flow diagram of the study is displayed in Fig. 1. The GSE46517 dataset containing gene expression data of 73 MM and 31 NMM samples was selected for bulk-seq analysis. Genes of the keratin family, such as KRT15 and KRT16, were up-regulated in NMM. In contrast, genes related to tumor metastasis, such as SPP1 [17] and CENPM [18], were up-regulated in MM (Fig. 2A, B). GO analysis, KEGG analysis, and GSEA revealed that the up-regulated genes in MM were enriched in metastasis-related pathways, including “positive regulation of Wnt signaling pathway” and “positive regulation of histone modification” (Fig. 2C, E) [19, 20]. Up-regulated genes in NMM were enriched in cell junction-related pathways, such as “cell-cell adhesion mediator activity,” “cell-matrix adhesion,” and “cell junction organization” (Fig. 2D, F). Previous studies reported that compared with NMM, the expression levels of SNAI2 [21–23], MMP2 [24–26], MIF [27–29], and AP1S2 [30] were up-regulated in MM and were crucial roles in the metastasis and malignant progression of melanoma. However, in the GSE46517 cohort, SNAI2 and MMP2 were down-regulated in MM (Fig. 2G). It was suspected that the characteristic of bulk-seq, which homogenizes all cells, may have contributed to this phenomenon. However, different tissues have different cellular compositions, which can obscure a significant amount of metastasis-related information. In general, the molecular landscapes of NMM and MM in GSE46517 were distinct, with MM being enriched in metastasis-related pathways. However, the limitations of bulk-seq may have led to some inaccuracies in the results.

### Clear evolutionary trajectory from NMM to MM at the single-cell level

**Fig. 3:**
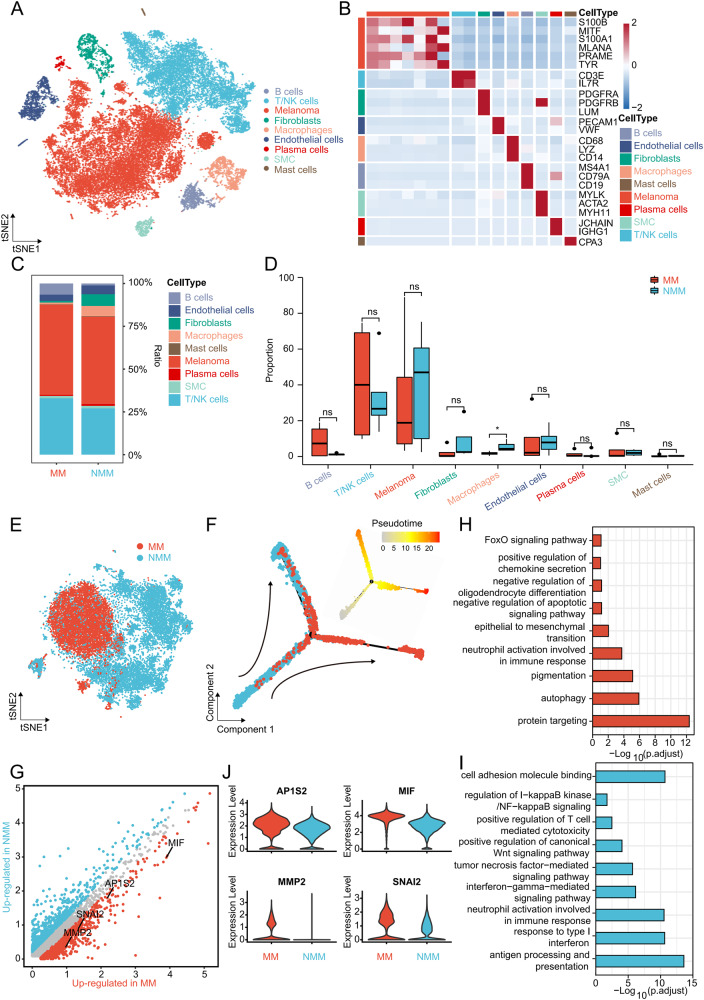
Plain evolutionary trajectory from NMM to MM at the single-cell level. **A** t-SNE plot showing the nine cell types annotated in the TME of melanoma. **B** Heatmap showing the marker genes of the nine cell types. **C**, **D** Bar (**C**) and box (**D**) plots comparing the different proportions of the nine cell types between NMM and MM. **E** t-SNE plot showing the melanoma cells of NMM and MM. **F** Pseudotime analysis revealing the evolutionary trajectory of melanoma cells. **G** Volcano plot depicting the up-regulated genes of NMM and MM at the single-cell level. **H**, **I** GO and KEGG analyses showing the enriched pathways and biological function in MM (**H**) and NMM (**I**). **J** Violin plots showing the expression levels of the four metastasis-related melanoma genes between MM and NMM at the single-cell level. NMM non-metastatic melanoma, MM metastatic melanoma, t-SNE t-distributed stochastic neighbor embedding, TME tumor microenvironment, GO Gene Ontology, KEGG Kyoto Encyclopedia of Genes and Genomes. *P < 0.05, **P < 0.01, ***P < 0.001, ****P < 0.0001.

GSE189889 contained scRNA-seq data of five NMM and four MM samples. After quality control and data preprocessing, all cells from the nine samples were grouped into 16 clusters (Supplemental Material Fig. 1A–C). Nine cell types, including melanoma, T/NK cells, fibroblasts, endothelial cells, macrophages, B cells, smooth muscle cells, plasma cells, and mast cells, were annotated using classical marker genes (Fig. 3A, B). The annotation of tumor cells, i.e., melanoma, was confirmed by the “inferCNV” package (Supplemental Material Fig. 1D). To explore the key cellular components involved in the metastasis of melanoma, cell–cell communication in NMM and MM was delineated and compared using the “CellChat” package. The results indicated that cell communication in MM was more tightly regulated than that in NMM (Supplemental Material Fig. 2A, B), characterized by higher interactions and stronger interaction strengths. In both NMM and MM, macrophages had the strongest incoming interaction strengths, while fibroblasts had the strongest outgoing interaction strengths (Supplemental Material Fig. 2C, F). The signaling patterns related to metastasis, such as ADGRE5 [31], PDGF [32], and SPP1 [33] were elevated in MM compared to NMM (Supplemental Material Fig. 2D, E, and S2G, H). The findings also indicated that receptor-ligand pairs contribute to metastasis, such as SPP1-CD44 [34, 35] and MDK-LRP1 [36], were elevated in MM (Supplemental Material Fig. 2I, J).

We further compared the differences in the proportions of each cell subtype between NMM and MM. A significantly lower proportion of macrophages was observed in MM (Fig. 3C, D, and Supplemental Material Fig. 1E). The macrophages were extracted for subpopulation analysis. Since all macrophages were derived from tumor tissues, we regarded them as tumor-associated macrophages (TAMs). All TAMs were downgraded into five clusters and further annotated as lipid-associated TAMs1 (LA-TAMs1), pro-angiogenic TAMs (Angio-TAMs), LA-TAMs2, immune regulatory TAMs (Reg-TAMs), and cytotoxic-TAMs based on previous findings [37] (Supplemental Material Fig. 3A–C). Enrichment analyses revealed that two LA-TAMs were enriched in “regulation of tumor necrosis factor production,” “response to lipid,” and “membrane lipid metabolic process”; Angio-TAMs were enriched in “positive regulation of cell migration”; Reg-TAMs were enriched in “regulation of CD8-positive, alpha-beta T cell activation”; and Cytotoxic-TAMs were enriched in “regulation of interleukin-2 production” confirming the annotation of macrophages (Supplemental Material Fig. 3D). The proportion of LA-TAMs1 and Angio-TAMs was decreased and increased, respectively, in MM tissues (Supplemental Material Fig. 3E). Finally, pseudotime analysis showed a clear evolutionary trajectory of TAMs from Reg-TAMs to Cytotoxic-TAMs to LA-TAMs1 to Angio-TAMs to LA-TAMs2 (Supplemental Material Fig. 3F), which demonstrated the malignant evolution during the process of metastasis.

To elucidate the mechanism of melanoma metastasis at a higher resolution, sub-cluster analysis was performed on melanoma cells (Fig. 3E). The pseudotime analysis revealed the evolutionary trajectory of melanoma cells from NMM to MM (Fig. 3F). GO and KEGG analyses demonstrated that metastasis-related pathways, such as the “epithelial to mesenchymal transition” and “autophagy,” were enriched in MM cells [38, 39], whereas “cell adhesion molecule binding” and “antigen processing and presentation” pathways were enriched in NMM cells (Fig. 3H, I). Finally, the expression levels of SNAI2, MMP2, MIF, and AP1S2 were elevated in melanoma cells of MM (Fig. 3G, J), consistent with the results of previous studies. The collective findings provided a comprehensive analysis of the TME and cell–cell communication of NMM and MM, revealing a clear evolutionary trajectory from NMM to MM at the single-cell level.

### Prognosis and metastatic potential of melanoma can be predicted by the MET score

**Fig. 4:**
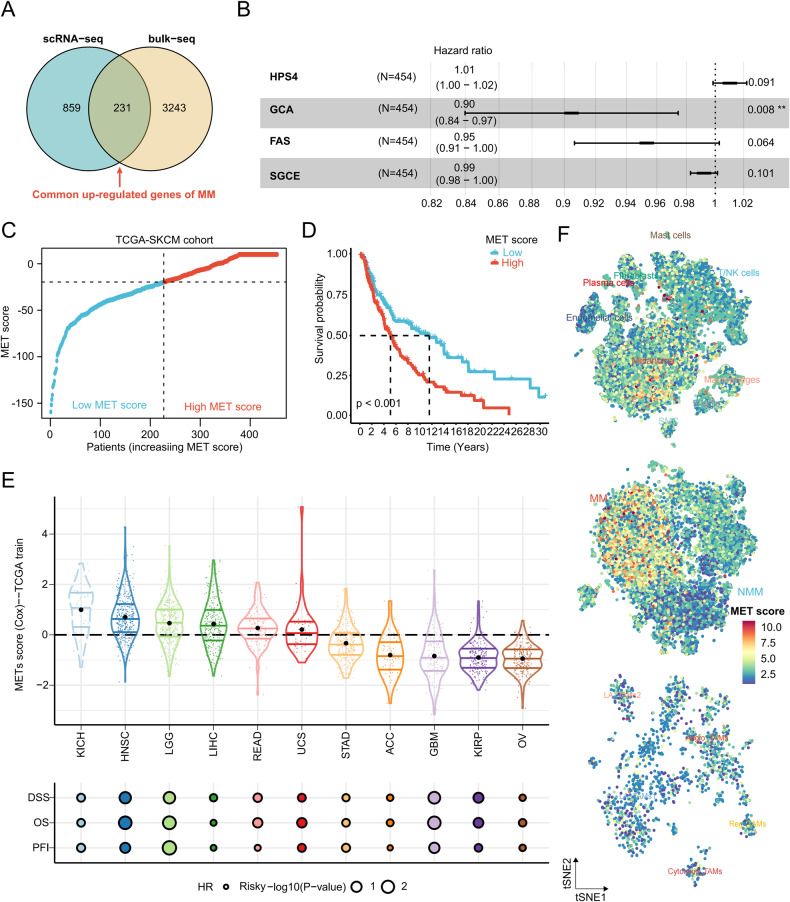
Predicting the metastatic potential and prognosis of melanoma by MET score. **A** Venn plot showing the common up-regulated genes in MM at bulk- and scRNA-seq levels. **B** Forest plot of four potential metastatic-related genes most related to the prognosis of melanoma screened by multivariate Cox regression analysis with a bootstrap algorithm. **C** Scatter plot showing the low and high MET score group in the TCGA-SKCM cohort. **D** Kaplan–Meier survival curves based on the MET score in the TCGA-SKCM cohort. **E** Validation of the prognosis prediction efficiency of the MET score in pan-cancers. **F** t-SNE plots showing the MET score in all cell types (upper panel), melanoma cells (middle panel), and macrophages (lower panel). MET score metastasis score, MM metastatic melanoma, bulk-seq bulk RNA sequencing, scRNA-seq single-cell RNA sequencing, t-SNE t-distributed stochastic neighbor embedding, TCGA-SKCM The Cancer Genome Atlas Skin Cutaneous Melanoma. *P < 0.05, **P < 0.01, ***P < 0.001, ****P < 0.0001.

Differential expression analysis was performed between NMM and MM at the bulk-seq level and between NMM cells and MM cells at the scRNA-seq level. A total of 231 commonly up-regulated genes in MM (i.e., potential metastatic-related genes) were screened from bulk- and scRNA-seq analyses (Fig. 4A, Supplemental Material Table 4). These genes were collected to establish the MET score. Univariate Cox regression analysis with a bootstrap algorithm, LASSO regression analysis, and multivariate Cox regression analysis with a bootstrap algorithm were performed, and four prognostic potential metastatic-related genes were identified for the establishment of the MET score (Supplemental Material Fig. 4A, B and Fig. 4B). The MET score was calculated using the aforementioned formula, and patients were stratified into low or high MET score groups based on the median MET score (Fig. 4C), indicating low or high metastatic potential, respectively. The survival analysis demonstrated that patients with high MET score had a worse OS compared to those with low MET score (Fig. 4D). The findings were validated in the GSE65904 cohort (Supplemental Material Fig. 4C, D). The MET score was confirmed as an independent prognostic factor for melanoma in the TCGA-SKCM cohort (Supplemental Material Fig. 4E). The subgroup analysis revealed that the MET score retained its prognostic prediction ability under different clinicopathological conditions (Supplemental Material Fig. 5). The MET score was also applied to the pan-cancer analysis, which revealed its consistent prognostic prediction effect in various types of cancer, such as kidney chromophobe, head and neck cancer, lower-grade glioma, and others (Fig. 4E). The “AddModuleScore” function of “Seurat” was used to calculate the MET score at the single-cell level. Angio-TAMs and melanoma cells, particularly MM cells, exhibited elevated MET scores compared to other cell types (Fig. 4F). In summary, the MET score was established and validated using bulk- and scRNA-seq data. The score may predict the metastatic potential and prognosis of melanoma.

### B16F10 cells display higher MET scores than B16F0 cells

**Fig. 5:**
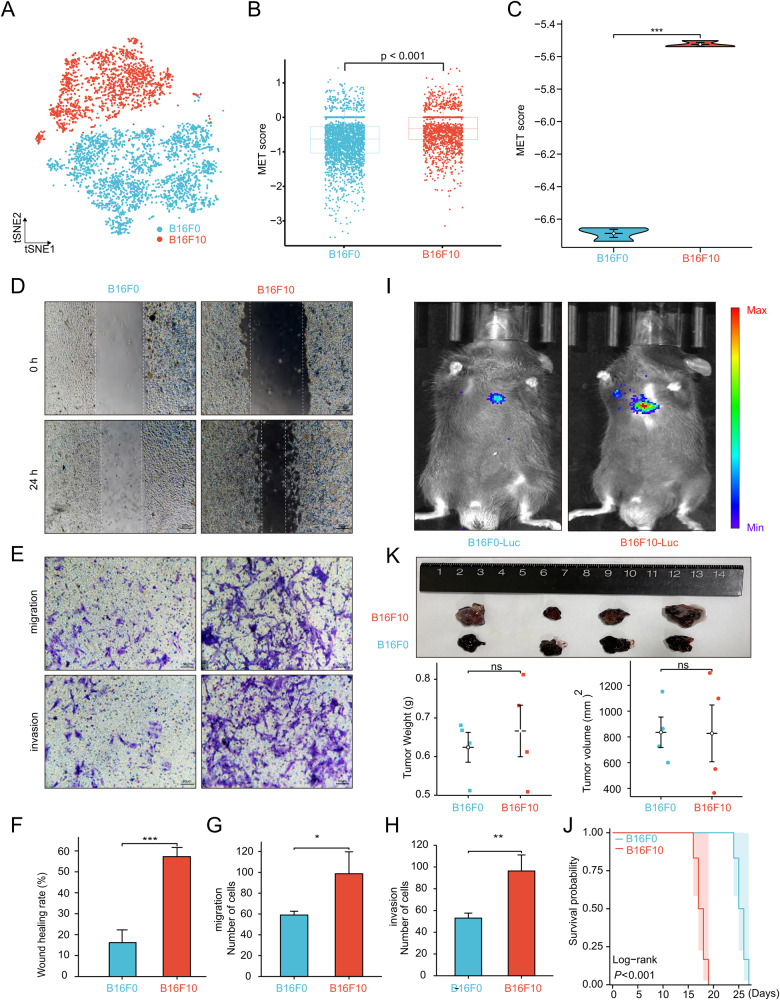
B16F0 and B16F10 could represent low and high MET score groups, respectively. **A** t-SNE plot showing the distribution of the B16F0 and B16F10 cells. **B** Comparison of MET score between B16F0 and B16F10 cells at the single-cell level. **C** Comparison of the MET score of B16F0 and B16F10 cells based on RNA-seq data. **D**, **E** Wound healing (**D**), Transwell migration (**E**, upper panel), and invasion (**E**, lower panel) assays of B16F0 and B16F10 cells. **F**–**H** Statistical charts of wound healing (**F**), migration (**G**), and invasion (**H**). **I** Bioluminescence assay showing the fluorescence intensity of the lung metastasis focuses on C57BL/6 mice injected with B16F0-Luc and B16F10-Luc cells. **J** Kaplan–Meier survival curves of subcutaneous xenograft tumor mice injected with B16F0 and B16F10 cells. **K** Subcutaneous xenograft tumor models showing the tumor weight and volume in C57BL/6 mice injected with B16F0 and B16F10 cells. t-SNE t-distributed stochastic neighbor embedding, MET score metastasis score. *P < 0.05, **P < 0.01, ***P < 0.001, ****P < 0.0001. All experiments were performed at least three times.

Based on the understanding that B16F0 serves as the parental cell line for B16 murine melanoma cells, while B16F10 is a highly metastatic subclone [40], it is plausible to hypothesize that the characteristics of B16F10 align with the high MET score group, whereas those of B16F0 correspond to the low MET score group. To confirm this hypothesis, scRNA-seq analysis of B16F0 and B16F10 was performed. As depicted in Fig. 5A, B16F0 and B16F10 cells exhibited clear distinctions, manifesting distinct transcriptome signatures. The MET score computed for each cell revealed a significantly higher MET score in B16F10 cells compared to B16F0 cells (Fig. 5B). The results of gene set variation analysis (GSVA) showed that B16F10 cells had higher t-value for “glycolysis,” “hypoxia,” and “Wnt/beta-catenin signaling,” whereas B16F0 had higher t-value of “myogenesis,” “DNA repair,” and “interferon-gamma response” (Supplemental Material Fig. 6A). The GO and KEGG analyses revealed that the up-regulated genes of B16F10 were enriched in metastasis-related pathways, such as “positive regulation of epithelial cell migration,” “HIF-1 signaling pathway,” and “PI3K-Akt signaling pathway” (Supplemental Material Fig. 6B) [41–43]. To confirm the characteristics of our B16F0 and B16F10 cells for further in vivo and in vitro assays, RNA-seq was conducted on both cell lines, and the MET score was recalculated based on the newly generated data. Our B16F10 cells also had a higher MET score than B16F0 cells at the RNA-seq level (Fig. 5C). GSEA revealed that B16F10 up-regulated genes were enriched in “epithelial-mesenchymal transition” and “JNK signaling” (Supplemental Material Fig. 6C) [38, 44].

In vitro assays were performed to validate the metastatic ability of B16F0 and B16F10 cells. Wound healing, migration, and invasion assays were performed, and the results consistently demonstrated that B16F10 cells exhibited a higher metastasis ability compared to B16F0 cells. This was evidenced by the increased migration and invasion capabilities of B16F10 cells, as observed in Fig. 5D–H.

In vivo assays were performed to assess the metastatic ability of B16F0 and B16F10 cells. A lung metastasis model was established by injecting 10^6^ B16F0-Luc or B16F10-Luc cells into the tail vein of 6-week-old C57BL/6 mice. Bioluminescence imaging revealed that mice injected with B16F10-Luc cells exhibited high fluorescence intensity (Fig. 5I), providing further evidence of the enhanced metastatic ability of B16F10 cells. Moreover, mice injected with B16F10-Luc cells displayed a worse OS compared to those injected with B16F0-Luc cells (Fig. 5J). Subcutaneous xenograft tumor models were also established, and no significant differences in tumor weight and volume were found between B16F0 and B16F10 cells (Fig. 5K). Collectively, the findings of the scRNA-seq and RNA-seq analyses and the in vitro and in vivo assays provided robust evidence supporting the high metastatic potential of B16F10 cells. These findings further substantiated the notion that B16F10 cells corresponded to the high MET score group and B16F0 cells corresponded to the low MET score group, suggesting their relevance as representative models for studying melanoma metastasis.

### Immunosuppressive microenvironment of the high MET score group may contribute to tumor metastasis

**Fig. 6:**
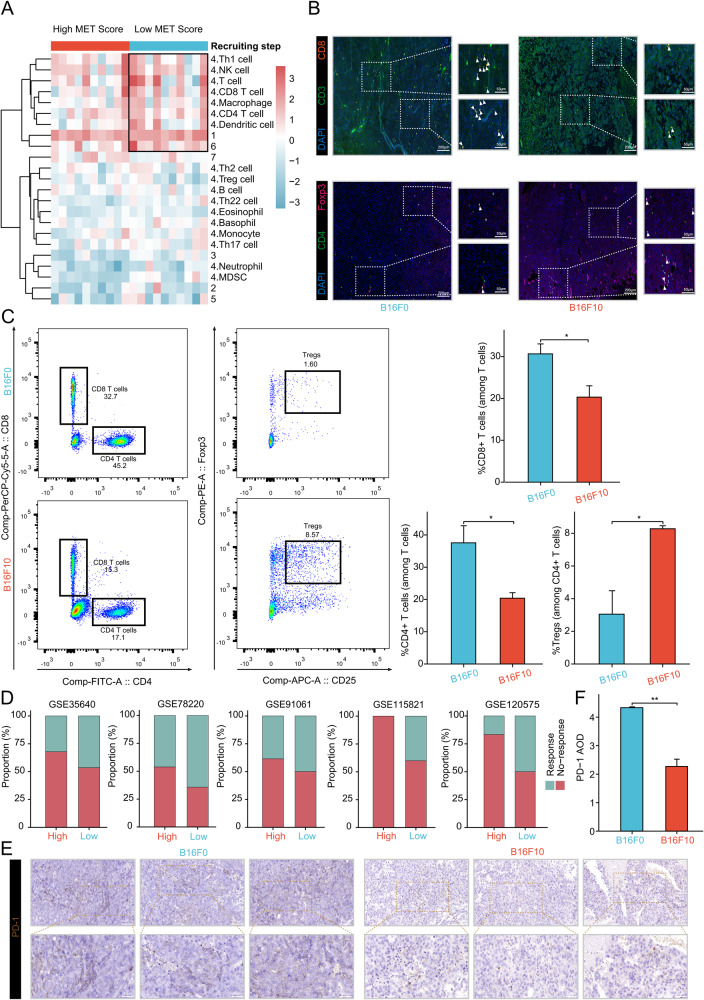
Different metastasis potential of the two MET score groups may result from different immune microenvironment. **A** TIP analysis revealing the different recruitment of immune cells in the two MET score groups. **B** Immunofluorescence assay showing the infiltration levels of CD8^+^ T cells and Tregs in the B16F0 and B16F10 induced subcutaneous xenografts. **C** Flow cytometry assay of infiltration levels of CD8 + T cells, CD4^+^ T cells, and Tregs in the B16F0 and B16F10 induced subcutaneous xenografts. **D** Different immunotherapy response rates of the two MET score groups in the four bulk-seq melanoma immunotherapy cohorts and one scRNA-seq melanoma immunotherapy cohort. **E** Immunohistochemical assay showing the different expression levels of PD-1 in the B16F0 and B16F10 induced subcutaneous xenografts. **F** Bar plot of the immunohistochemical assay. MET score metastasis score, TIP tracking tumor immunophenotype, bulk-seq bulk RNA sequencing, scRNA-seq single-cell RNA sequencing. *P < 0.05, **P < 0.01, ***P < 0.001, ****P < 0.0001. All experiments were performed at least three times.

Previous studies have reported the pivotal role of immune cell interactions within the tumor ecosystem in influencing both metastasis and the response to immunotherapy in patients [45–47]. Therefore, we hypothesized that distinct differences in the immune microenvironment exist between the two MET score groups. Utilizing transcriptome data and CIBERSORT analysis, we observed upregulation of resting mast cells and downregulation of gamma delta T cells in the high MET score group within the TCGA-SKCM cohort. Furthermore, in the GSE65904 cohort, the high MET score group displayed up-regulated expression of Tregs (Supplemental Material Fig. 7A, B). TIP analysis further supported these observations; the low MET score group exhibited higher recruitment of antitumor immune cells, including CD8^+^ T cells and NK cells (Fig. 6A). These findings suggest an immunosuppressive microenvironment associated with the high MET score group that potentially contributes to tumor metastasis. Immunofluorescence and flow cytometry results confirmed that CD8^+^ T cells were down-regulated and Tregs were up-regulated in the B16F10 induced subcutaneous xenograft TME (Fig. 6B, C). To explore the potential impact of the MET score on immunotherapy response, the expression levels of common immune checkpoints were compared between the low and high MET score groups in the TCGA-SKCM cohort. Intriguingly, the analysis of immune checkpoint expression in the cohort revealed elevated levels of CD274, CTLA4, LAG3, PDCD1 (encoding PD-1 protein), and TIGIT in the low MET score group (Supplemental Material Fig. 7C). A prior analysis of 46 melanoma samples treated with aPD-1 therapy revealed significantly higher expressions of PD-1 and PD-L1 in the response group compared to the no-response group [48]. Thus, we suspect that the low MET score group may have a higher likelihood of responding to aPD-1 compared to the high MET score group. Four bulk-seq melanoma immunotherapy cohorts were used to construct the MET score; the results confirmed our hypothesis (Fig. 6D). To further assess the efficacy of the MET score in predicting immunotherapy response, we extended our analysis to a scRNA-seq cohort with available data on aPD-1 response in melanoma patients. The results consistently demonstrated that individuals belonging to the low MET score group exhibited a notably higher response rate to aPD-1 in comparison to those categorized within the high MET score group (Fig. 6D, and Supplemental Material Fig. 8A–C). These findings support the hypothesis that the immune microenvironment associated with the low MET score group may be more conducive to immunotherapy efficacy in melanoma patients. Immunohistochemical assays were performed to further investigate the expression level of PD-1, an important immune checkpoint commonly targeted in melanoma immunotherapy. The results confirmed higher expression of PD-1 in the TME of the B16F0 induced subcutaneous xenograft model (Fig. 6E, F). Taken together, the immunosuppressive microenvironment of the high MET score group and higher expression levels of PD-1 in the low MET score group suggest that the low MET score group, represented by the B16F0 cells, may exhibit a higher potential for PD-1/PD-L1 pathway-mediated immunotherapy response.

### Monotherapy of aPD-1 effectively inhibits metastasis of melanoma in the low MET score group

**Fig. 7:**
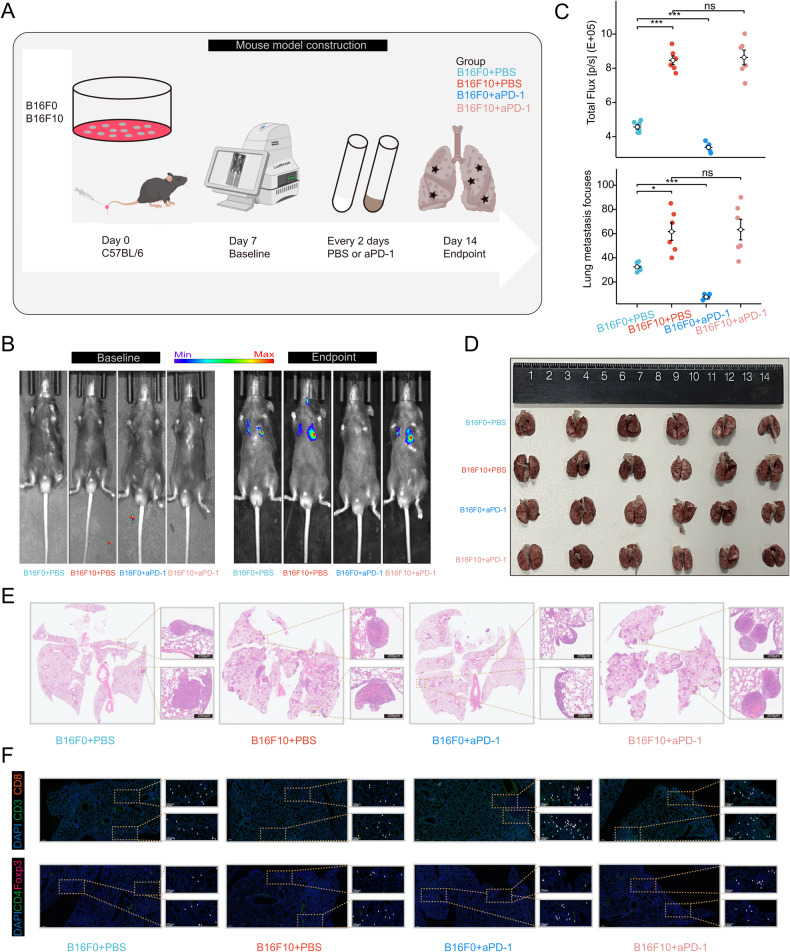
Early intervention with aPD-1 significantly inhibits lung metastasis in the low MET score group. **A** Workflow of the lung metastasis model. C57BL/6 mice were divided into four groups, namely, B16F0-PBS, B16F10-PBS, B16F0-aPD-1, and B16F10-aPD-1 (*n* = 6 per group), and B16F0-Luc or B16F10-Luc cells were injected into the tail vein. **B** Bioluminescence assay showing the fluorescence intensity of lung metastasis focuses on C57BL/6 mice at baseline (left panel) and endpoint (right panel). **C** Statistical charts of the bioluminescence assay (upper panel) and H&E staining assay (lower panel). **D** Image showing the dissected lungs of C57BL/6 mice in these four groups. **E** Representative H&E images of lung metastasis focuses. **F** Immunofluorescence assay showing the infiltration levels of CD8^+^ T cells and Tregs in the lung metastasis focuses of the four groups. aPD-1 anti-PD-1, MET score metastasis score, H&E hematoxylin and eosin. *P < 0.05, **P < 0.01, ***P < 0.001, ****P < 0.0001. All experiments were performed at least three times.

Lung metastasis mouse models were constructed to investigate whether the low MET score group exhibited a higher response rate to aPD-1 compared to the high MET score group. C57BL/6 mice were randomly divided into four groups: B16F0-PBS, B16F10-PBS, B16F0-aPD-1, and B16F10-aPD-1 (*n* = 6 per group) (Fig. 7A). At baseline, the bioluminescence assay confirmed the absence of lung metastasis in all four groups (Fig. 7B). At the endpoint, a significant decrease in fluorescence intensity was observed in the B16F0-aPD-1 group compared to the B16F0-PBS group, indicating that aPD-1 monotherapy effectively inhibited lung metastasis in the low MET score group. However, no significant difference in fluorescence intensity was observed between the B16F10-PBS and B16F10-aPD-1 groups, suggesting that aPD-1 treatment did not significantly impact lung metastasis in the high MET score group (Fig. 7B, C). The mice were euthanized, and the lungs were subsequently removed for hematoxylin and eosin (H&E) staining and immunofluorescence assays (Fig. 7D). H&E staining confirmed that treatment with aPD-1 significantly reduced the number of lung metastatic foci in mice injected with B16F0 cells. However, no significant difference was observed in mice injected with B16F10 cells after aPD-1 treatment (Fig. 7C, E). Lastly, the immune microenvironment within the lung metastatic foci of the four groups was investigated. The aPD-1 treatment increased the infiltration of CD8^+^T cells in the B16F0 groups. However, no significant changes were observed in the infiltration of Tregs after aPD-1 treatment (Fig. 7F). In general, the high response rate to aPD-1 was verified in the low MET score group based on the mouse lung metastasis models.

## Discussion

Metastasis plays a pivotal role in the progression of melanoma and is strongly associated with reduced patient survival [1, 49]. Accurate prediction of metastatic potential in resected NMM and the selection of suitable postoperative adjuvant treatments to prevent metastasis are crucial in improving patient prognosis. Immunotherapy, particularly the use of ICIs, has significantly enhanced the prognosis of advanced and unresectable MM [4, 50, 51]. However, the efficacy of ICIs in preventing the development of metastases in resected NMM remains contentious and requires further investigation. American Society of Clinical Oncology guidelines do not currently recommend adjuvant pembrolizumab and nivolumab for patients with resected stage II melanoma, except in the context of clinical trials [9]. However, when used as adjuvant therapy, toripalimab exhibited comparable distant metastasis-free survival and improved safety compared to high-dose interferon-α2b in patients with resected mucosal melanoma [8]. In the present study, we developed and validated the MET score using cross-species bulk-seq and scRNA-seq data and in vitro and in vivo experiments. Our findings reveal that the MET score serves as a robust prognostic indicator and accurately predicts the metastatic potential of melanoma. The MET score is also a valuable tool in determining the response rate to aPD-1 immunotherapy in melanoma patients. Thus, the MET score holds great promise as a powerful resource for guiding effective therapeutic strategies in managing melanoma.

First, metastasis of melanoma was investigated using bulk-seq, shedding light on the underlying mechanisms. An intriguing discrepancy was observed in the expression levels of two well-established metastasis-related genes, SNAI2 and MMP2, in MM samples from the GSE46517 dataset [21–26]. This discrepancy highlights the limitations of bulk-seq in fully capturing the complex dynamics of melanoma metastasis. To gain a comprehensive understanding, it is imperative to employ high-resolution sequencing technologies that can provide a more nuanced view of the metastatic process. To reconcile this discrepancy, scRNA-seq analysis was conducted on both NMM and MM samples. Subpopulation analysis of TAMs revealed distinct changes in the composition of TAM subsets in MM compared to NMM. Specifically, the proportion of LA-TAMs1 decreased, and Angio-TAMs increased in MM. LA-TAMs have been implicated in promoting the inflammatory response and immune system activation, while LA-TAMs associated with lipid catabolism exhibit immunosuppressive properties and tolerance [52, 53]. On the other hand, Angio-TAMs are characterized by high expression of angiogenic genes and can facilitate metastasis by promoting tumor cell intravasation and extravasation [54, 55]. Pseudotime analysis revealed a progressive transition from an anti- to pro-tumor phenotype in TAMs. Furthermore, the expression levels of four previously identified metastasis-related genes were compared at single-cell resolution. Interestingly, all four genes were up-regulated in MM, providing a clear resolution to the contradiction observed in the bulk-seq analysis. This finding highlights the power of scRNA-seq in capturing cellular heterogeneity and elucidating the intricate mechanisms underlying melanoma metastasis.

In addition, by leveraging the shared up-regulated genes in MM and employing various machine-learning algorithms, we successfully developed the MET score as a predictive tool for assessing the metastatic potential of melanoma. The validity of the MET score was further confirmed through pan-cancer and scRNA-seq analyses, which demonstrated the ability of the MET score to accurately predict both metastatic potential and prognosis in patients with various types of cancer.

Based on previous findings that highlighted the highly metastatic nature of B16F10 as compared to B16F0 [40], it was hypothesized that B16F10 cells would exhibit a higher MET score compared to B16F0 cells. Consequently, these two cell lines were considered suitable representatives of the low and high MET score groups, respectively. This hypothesis was confirmed through comprehensive scRNA-seq and RNA-seq analyses. In addition, a series of functional assays, including wound healing, invasion, migration, and in vivo tumor metastasis models, were conducted. The data unequivocally demonstrated the enhanced metastatic potential of B16F10 cells compared to B16F0 cells. These collective findings robustly support using these two cell lines as representative models for characterizing the high and low MET score groups. Given the well-established impact of the TME on metastasis and response to ICIs in melanoma patients [46, 47, 56], it was postulated that differences in the immune microenvironment between the two MET score groups would exist. Using the advanced analytical techniques of CIBERSORT and TIP, we observed that the high MET score group exhibited an immunosuppressive microenvironment characterized by elevated infiltration levels of Tregs and reduced infiltration levels of CD8^+^ T cells compared to the low MET score group. To validate these findings, immunofluorescence and flow cytometry assays were performed. The findings confirmed the previous observations, revealing the upregulation of Tregs and downregulation of CD8^+^ T cells within the TME induced by B16F10 cells in the subcutaneous xenograft model. These results further support the notion of an immunosuppressive microenvironment associated with the high MET score group, suggesting a potential mechanism underlying the enhanced metastatic potential of B16F10 cells. As ICIs have become a cornerstone of melanoma immunotherapy, it was crucial to assess the expression levels of common immune checkpoints in relation to the MET score groups. Interestingly, in the low MET score group, there was a notable elevation in the expression levels of several immune checkpoints, including CD274, CTLA4, LAG3, PDCD1, and TIGIT. These findings suggest that patients within the low MET score group may exhibit a more favorable response to immunotherapy. Thus, the MET score in melanoma immunotherapy cohorts of bulk- and scRNA-seq levels was determined. Unexpectedly, the low MET score group showed a higher response rate to immunotherapy. Considering that PD-1 is the predominant target in melanoma immunotherapy, various PD-1 inhibitors, including nivolumab [57, 58], pembrolizumab [59–61], and toripalimab [62, 63], have been extensively utilized in clinical settings for MM treatment. PD-1 was chosen for further investigation and analysis. Immunohistochemistry verified the higher expression of PD-1 in the B16F0 induced subcutaneous xenograft TME. Previous study had reported the association between PD-1 expression levels and aPD-1 response rate [48]. We hypothesized that aPD-1 could effectively inhibit the metastasis of resected NMM in the low MET score group, thus serving as a promising adjuvant therapy.

To further validate the hypothesis that aPD-1 can effectively inhibit postoperative metastasis in the low MET score group, we conducted in vivo experiments using tail vein lung metastasis mouse models. At baseline, bioluminescence assay results confirmed the absence of lung metastases in all four groups of mice. However, at the endpoint, a notable decrease in fluorescence intensity and a significant reduction in the number of lung metastasis foci were observed in the B16F0-aPD-1 group compared to the B16F0-PBS group. In contrast, no significant difference was observed between the two B16F10 groups. These findings strongly support the view that aPD-1 treatment is highly effective in inhibiting postoperative metastasis in the low MET score group. Finally, immunofluorescence analysis revealed that treatment with aPD-1 led to enhanced infiltration of CD8^+^ T cells in the TME of lung metastases in the B16F0 groups. These findings indicate better responsiveness to aPD-1 of the low MET score groups compared to the high MET score group. Thus, aPD-1 is recommended as a postoperative adjuvant treatment strategy to reduce the risk of metastasis in NMM with a low MET score, indicative of lower metastatic potential. For patients with a high MET score, indicating a higher metastatic potential, the combination of high-dose interferon-α2b as a potential treatment option may be warranted.

One limitation of our study is that due to the rarity of melanoma, we could not expand the MET score to our melanoma cohort. However, to address this challenge, we validated the MET score using large publicly available melanoma cohorts at both the bulk- and scRNA-seq levels. This approach allowed us to leverage the existing data from a broader population and strengthened the robustness and generalizability of our findings.

## Conclusions

We successfully developed and validated the MET score using a comprehensive approach that incorporates cross-species bulk- and scRNA-seq data, as well as in vivo and in vitro assays. The MET score is a reliable predictor of the metastatic potential and prognosis of melanoma. For patients with a low MET score, indicating a lower metastatic potential, we recommend using aPD-1 as a postoperative adjuvant treatment to effectively reduce the risk of metastasis following resection. A combination therapy approach might be more suitable for patients with a high MET score, suggesting a higher metastatic potential.

## Materials and methods

### Data sources

Data from different sources were utilized to develop and validate the MET score. Bulk- and scRNA-seq data for NMM and MM were obtained from the GSE46517 and GSE189889 datasets, respectively. To establish the MET score, gene expression, clinical features, and survival data were downloaded from The Cancer Genome Atlas Skin Cutaneous Melanoma (TCGA-SKCM) cohort. External validation was performed using the GSE65904 cohort, which includes gene expression and survival data for melanoma. Pan-cancer transcriptome data were obtained from the Xena visual integration and exploration tool [64]. In addition, scRNA-seq data of the B16F0 and B16F10 cell lines were obtained from the GSE156444 dataset. RNA-seq data of our B16F0 and B16F10 cell lines are provided in Supplemental Material Table 1. Four melanoma bulk-seq cohorts (GSE35640, GSE78220, GSE91061, and GSE115821) and one melanoma scRNA-seq cohort (GSE120575) with immunotherapy response data were used to test the efficiency of the MET score in predicting immunotherapy responses. Taken into consideration the impact of any anti-tumor drug therapy on gene expression patterns, we exclusively retained samples from patients who had not received prior targeted therapy or immunotherapy before surgery for the construction of the MET score.

### Bulk-seq analysis

Differential expression analysis was performed between NMM and MM using “DESeq2,” and the results were visualized using the R packages “ggplot2” and “ComplexHeatmap” [65]. Gene Ontology (GO), Kyoto Encyclopedia of Genes and Genomes (KEGG), and gene set enrichment analysis (GSEA) revealed specific pathways enriched in NMM and MM using the “clusterprofiler” package.

### scRNA-seq analysis

In the quality control stage, only cells with >200 detected genes and <20% of mitochondrial genes were retained. Cell cycle effect was considered by the “CellCycleScoring” function of the “Seurat” package.

The “NormalizeData,” “FindVariableFeatures,” “ScaleData,” and “RunPCA” functions of the “Seurat” package were used to preprocess the data. The “harmony” package was used for batch-effect removal. Graph-based clustering and t-distributed stochastic neighbor embedding (t-SNE) were performed for visualization.

Using the “FindAllMarkers” function, the marker genes of each cluster were identified. The cells were manually annotated using classical marker genes (Supplemental Material Table 2). The “infercnv” package was used to confirm the annotation of tumor cells based on chromosome copy number alterations. The “CellChat” package was used for cell–cell communication analysis. Subsequently, macrophages and tumor cells were extracted separately for sub-cluster analyses, and the aforementioned data preprocessing procedures were performed. Pseudotime analysis was performed to reveal the cell evolution process by the “monocle” package. Finally, GO and KEGG analyses demonstrated enriched pathways in NMM and MM at the single-cell resolution.

### Construction of the MET score

Univariate Cox regression analysis with a bootstrap algorithm, least absolute shrinkage and selection operator (LASSO) regression analysis, and multivariate Cox regression analysis with a bootstrap algorithm were performed to screen the potential metastatic-related genes that were highly correlated with OS. The bootstrap coefficient of the included potential metastatic-related gene was defined using the formula: bootstrap coefficient = $\tfrac{{\rm{coefficient}}}{{\rm{bootstrap}}\,{\rm{standard}}\,{\rm{deviation}}}$ [66]. The MET score was obtained using the following formula: MET score = ${\sum }_{i=1}^{n}{\rm{bootstrap}}\,{\rm{coefficient}}\,({\rm{included}}\,{\rm{potential}}\,{\rm{metastatic}}-{\rm{related}}\,{{\rm{gene}}}_{i})\times {\rm{expression}}\,{\rm{level}}\,({\rm{included}}\,{\rm{potential}}\,{\rm{metastatic}}-{\rm{related}}\,{{\rm{gene}}}_{i}).$

### Immune microenvironment analysis

CIBERSORT was used to quantify the immune microenvironment using the deconvolution algorithm in the TCGA-SKCM and GSE65904 cohorts [67, 68]. Tracking of the tumor immunophenotype (TIP) was used to explore the anticancer immune status of the two groups based on the tumor immune cycle in seven stages [69].

### Cell culture and transfection

The B16F0 (RRID: (CVCL_0604)) and B16F10 (RRID: (CVCL_0159)) cell lines purchased from the Cell Bank of the Chinese Academy of Sciences (Shanghai, China) were cultured as previous reported [70]. CMV-Luc-Puro lentiviral vectors (Genomeditech, Shanghai, China) were transfected into B16F0 and B16F10 cells and used for the bioluminescence assay. B16F0-Luc and B16F10-Luc cells were filtered using puromycin (2 mg/mL; Yeasen, Shanghai, China) for 10 days to establish stable cell lines. All experiments were performed with mycoplasma-free cells.

### RNA-seq of B16F10 and B16F10 cells

RNA-seq was performed on the B16F10 and B16F10 cell lines. Briefly, RNA was extracted using the TRIzol reagent (Invitrogen, Carlsbad, CA, USA). An mRNA library was constructed by Genergy Bio-Technology (Shanghai, China). The library was sequenced using a Novaseq6000 apparatus (Illumina, San Diego, CA, USA). The reads were aligned to the CRCh38 human reference genome. The “edgeR” package was used for normalization, and the expressions of RNA were transformed to fragments per kilobase of transcript per million mapped reads for further analysis.

### Wound healing, invasion, and migration assays

For the wound healing assay, when cells reached confluence, artificial wounds were created by scratching the growth with a sterile 200 μL pipette tip. Photographs were taken at 0 and 24 h using a camera attached to a light microscope (Olympus, Tokyo, Japan). For the invasion assay, Matrigel (BD Biosciences, Franklin Lakes, NJ, USA) was equally spread in a Transwell chamber and inoculated with 10^4^ cells in 200 μL of serum-free Dulbecco’s modified eagle medium. This was followed by the addition of 600 μL of medium containing 20% fetal bovine serum (FBS) to the lower chamber. After 60 h, the chambers were fixed in 4% paraformaldehyde (Biosharp, Guangzhou, China) and stained with crystal violet (Yeasen). Samples were examined by light microscope (Olympus) and photographed. For the migration assay, the same protocol was followed, except that Matrigel was not added, and the time of cell culture was adjusted to 24 h.

### In vivo assays

A schematics diagram representing the workflow was created using Figdraw (https://www.figdraw.com/static/index.html/). For the metastasis model, 1 × 10^6^ B16F0-Luc or B16F10-Luc cells were injected into the tail vein of 6-week-old C57BL/6 mice (*n* = 6 per group). Bioluminescence assays were performed by injecting 200 μL D-luciferin potassium salt (15 mg/mL; Yeasen) into the enterocoelia. The fluorescence intensity of lung metastases was observed using an IVIS Lumina III apparatus (PerkinElmer, Waltham, MA, USA). The survival status of the mice was recorded daily, and survival analysis was performed.

Subcutaneous xenograft tumor models of B16F0 and B16F10 cells were also established by injecting 5 × 10^6^ cells per mouse into the armpits of C57BL/6 mice (*n* = 4 per group). The mice were sacrificed 20 days after injection. Tumors were weighed and measured (volume = *L* × *W*^2^ × 0.5, where *L* and *W* represent the largest and smallest diameters, respectively).

### Immunofluorescence, flow cytometry, and immunohistochemical assays

An immunofluorescence assay was used to detect the infiltration levels of CD8^+^ T cells and regulatory T cells (Tregs) in the TME. Cells were fixed with 4% paraformaldehyde, incubated with 0.3% Triton X-100 (Yeasen), blocked with 5% FBS, and incubated with the primary antibody at 4 °C overnight, followed by incubation with the appropriate secondary antibody. Nuclei were counterstained with 4’,6-diaminoamino-2-phenylindole (Yeasen). Fluorescence intensity was detected by confocal laser scanning microscope (Zeiss, Oberkochen, Germany).

Flow cytometry was performed to confirm the infiltration levels of CD8^+^ T cells and Tregs in the TME. Subcutaneous xenograft tumors were harvested after the mice were euthanized. Tumors were digested with collagenase IV (Sigma-Aldrich, St. Louis, MO, USA) and DNase I (Sigma-Aldrich). For fluorochrome-labeled membrane markers, single-cell suspensions were resuspended in phosphate-buffered saline (PBS) (Genom, Jiaxin, China) with 0.1% bovine serum albumin (Gibco, Carlsbad, CA, USA) and then stained. For fluorochrome-labeled intranuclear markers, single-cell suspensions were treated with Fixation/Permeabilization Solution Kit (BD Biosciences, Franklin Lakes, NJ, USA) and then stained. Data were collected using BD Fortessa X20 (BD Biosciences) and analyzed using FlowJo V10 software (https://www.flowjo.com/solutions/flowjo/downloads).

Immunohistochemical assays were performed to identify the expression levels of PD-1 in tumor tissues. The slides were baked, dewaxed, and rehydrated. Antigen extraction was performed after incubation with 0.3% H_2_O_2_. The sections were incubated with primary and secondary antibodies at 4 °C overnight. The sections were then stained with diaminobenzidine (Sigma-Aldrich), counterstained with hematoxylin (Yeasen), dehydrated with ethanol (Yeasen), cleared with xylene (Sigma-Aldrich), and covered with resin (Yeasen).

All antibodies are listed in Supplemental Material Table 3.

### Statistical analyses

Statistical analyses, including student’s t-test, Wilcoxon rank-sum test, one-way analysis of variance, log-rank test, and Cox regression analyses, were performed using R 4.1.1 (R foundation for statistical computing, Vienna, Austria). Unless otherwise stated, the statistical significance cut-off value was set at *P* < 0.05.

### Supplementary information

Spplementary figure and table legends

Supplemental Material Figure 1

Supplemental Material Figure 2

Supplemental Material Figure 3

Supplemental Material Figure 4

Supplemental Material Figure 5

Supplemental Material Figure 6

Supplemental Material Figure 7

Supplemental Material Figure 8

Supplemental Material Table 1

Supplemental Material Table 2

Supplemental Material Table 3

Supplemental Material Table 4

## Data Availability

RNA-seq data of B16F0 and B16F10 cells are available on Supplemental Material Table 1.
