# Supplementary material for: Decoding the metastatic potential and optimal postoperative adjuvant therapy of melanoma based on metastasis score
Source: Cell Death Discov. 2023 Oct 25;9:397. doi: 10.1038/s41420-023-01678-6 (PMC10600209; doi:10.1038/s41420-023-01678-6)
Supplement: Supplementary file 1 — Spplementary figure and table legends [file 41420_2023_1678_MOESM1_ESM.docx]

**Supplemental Material Figure 1. Decoding the metastasis of melanoma at the single-cell level.** (A) t-SNE plot showing that all cells could be divided into 16 clusters. (B) t-SNE plot showing the cellular landscape based on the sample source. (C) t-SNE plot showing the cellular landscape based on the tissue type. (D) InferCNV analysis confirms the annotation of melanoma cells. (E) Box plot comparing the different proportions of the nine cell types in each sample. t-SNE, t-distributed stochastic neighbor embedding.

**Supplemental Material Figure 2. CellChat analysis showing the different cell–cell communications between NMM and MM.** (A) The cell–cell interaction levels of NMM and MM. The red curve denotes increased cell–cell interactions in MM and the blue curve denotes increased cell–cell interactions in NMM. (B) Cell–cell interaction strength of NMM and MM. The red curve denotes increased cell–cell interaction strength in MM and the blue curve denotes increased cell–cell interaction strength in NMM. (C) Outgoing and incoming interaction strength of the nine cell types in NMM. (D-E) Heat maps showing the (D) outgoing and (E) incoming signaling patterns of NMM. The height of the top bar plot denotes the interaction strength of the corresponding cell type, and the height of the bar on the right denotes the strength of the signaling pattern. (F) Outgoing and incoming interaction strength of the nine cell types in MM. (G-H) Heat maps showing the outgoing (G) and incoming (H) signaling patterns of MM. The height of the top bar plot denotes the interaction strength of the corresponding cell type. The height of the bar on the right denotes the strength of the signaling pattern. (I-J) Bubble plots showing the increased (I) and decreased (J) ligand-receptor pairs from melanoma cells to other cells in MM compared to NMM. NMM, non-metastatic melanoma; MM, metastatic melanoma.

**Supplemental Material Figure 3. Subpopulation analysis showing the evolutionary trajectory of macrophages during melanoma metastasis.** (A) t-SNE plot showing that all macrophages could be divided into five types. (B) Scatter plot showing the up- and down-regulated genes in the five types of macrophages. (C) Heat map showing the expression levels of the annotation genes of the five types of macrophages. (D) GO and KEGG analyses depict the specific enriched pathways and biological function of the five types of macrophages. (E) Bar plot comparing the different proportions of the five types of macrophages between NMM and MM. (F) Pseudotime analysis revealing the evolutionary trajectory of the five types of macrophages. t-SNE, t-distributed stochastic neighbor embedding; GO, Gene Ontology; KEGG, Kyoto Encyclopedia of Genes and Genomes; NMM, non-metastatic melanoma; MM, metastatic melanoma.

**Supplemental Material Figure 4. Development and validation of the MET score by machine learning algorithms.** (A) Validation of the prognostic potential metastatic-related genes using LASSO regression analysis. (B) Explanation for the LASSO coefficient profile plot of prognostic potential metastatic-related genes. (C) Scatter plot showing the low and high MET score group in the GSE65904 cohort. (D) Kaplan-Meier survival curves based on the MET score in the GSE65904 cohort. (E) Forest plot showing the independent prognostic analysis of the MET score in the TCGA-SKCM cohort. MET score, metastasis score; LASSO, least absolute shrinkage, and selection operator; TCGA-SKCM, The Cancer Genome Atlas Skin Cutaneous Melanoma.

**Supplemental Material Figure 5. Subgroup survival analysis of the MET score.** MET score, metastasis score.

**Supplemental Material Figure 6. Enrichment analyses of B16F0 and B16F10 cells.** (A) GSVA depicting the enrichment score of 12 gene lists of B16F0 and B16F10 cells based on single-cell data. (B) GO and KEGG analyses showing the enriched pathways and biological function of B16F10 cells based on single-cell data. (C) GSEA depicting the enriched gene lists of B16F0 and B16F10 cells based on RNA-seq data. GSVA, gene set variation analysis; GO, Gene Ontology; KEGG, Kyoto Encyclopedia of Genes and Genomes; GSEA, gene set enrichment analysis.

**Supplemental Material Figure 7. Immune microenvironment analysis of the two MET score groups.** (A-B) The infiltration levels of 22 immune cell types between the two MET score groups in TCGA-SKCM (A) and GSE65904 (B) cohorts based on CIBERSORT. (C) Expression levels of immune checkpoints between the two MET score groups in the TCGA-SKCM cohort. MET score, metastasis score, TCGA-SKCM, The Cancer Genome Atlas Skin Cutaneous Melanoma. * P < 0.05, ** P < 0.01, *** P < 0.001, **** P < 0.0001.

**Supplemental Material Figure 8. Construction of the MET score in GSE120575 cohort.** (A-C) t-SNE plots illustrating the distribution of samples (A), immunotherapy response types (B), and MET score (C) in GSE120575. MET score, metastasis score; t-SNE, t-distributed stochastic neighbor embedding.

**Supplemental Material Table 1. RNA-seq of B16F0 and B16F10.**

**Supplemental Material Table 2. The marker genes for annotation.**

**Supplemental Material Table 3. Antibodies used for immunofluorescence, flow cytometry, immunohistochemistry, and in vivo assays.**

**Supplemental Material Table 4. Common up-regulated genes of in metastatic melanoma at bulk- and single-cell levels.**
