# Supplementary figures and images for: Decoding the metastatic potential and optimal postoperative adjuvant therapy of melanoma based on metastasis score
Source: Cell Death Discov. 2023 Oct 25;9:397. doi: 10.1038/s41420-023-01678-6 (PMC10600209; doi:10.1038/s41420-023-01678-6)

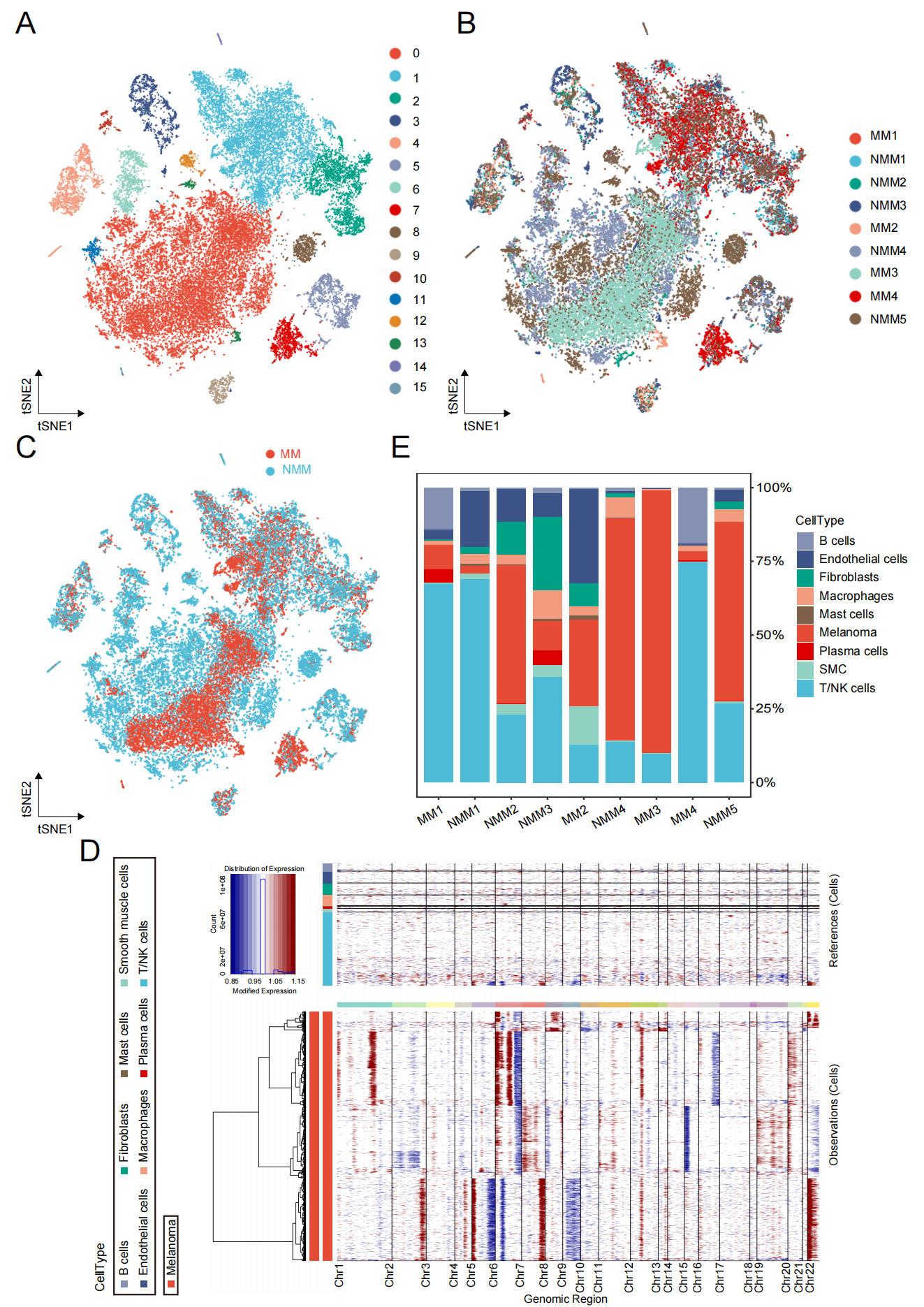

Supplement: Supplementary file 2 — Supplemental Material Figure 1 [file 41420_2023_1678_MOESM2_ESM.png]

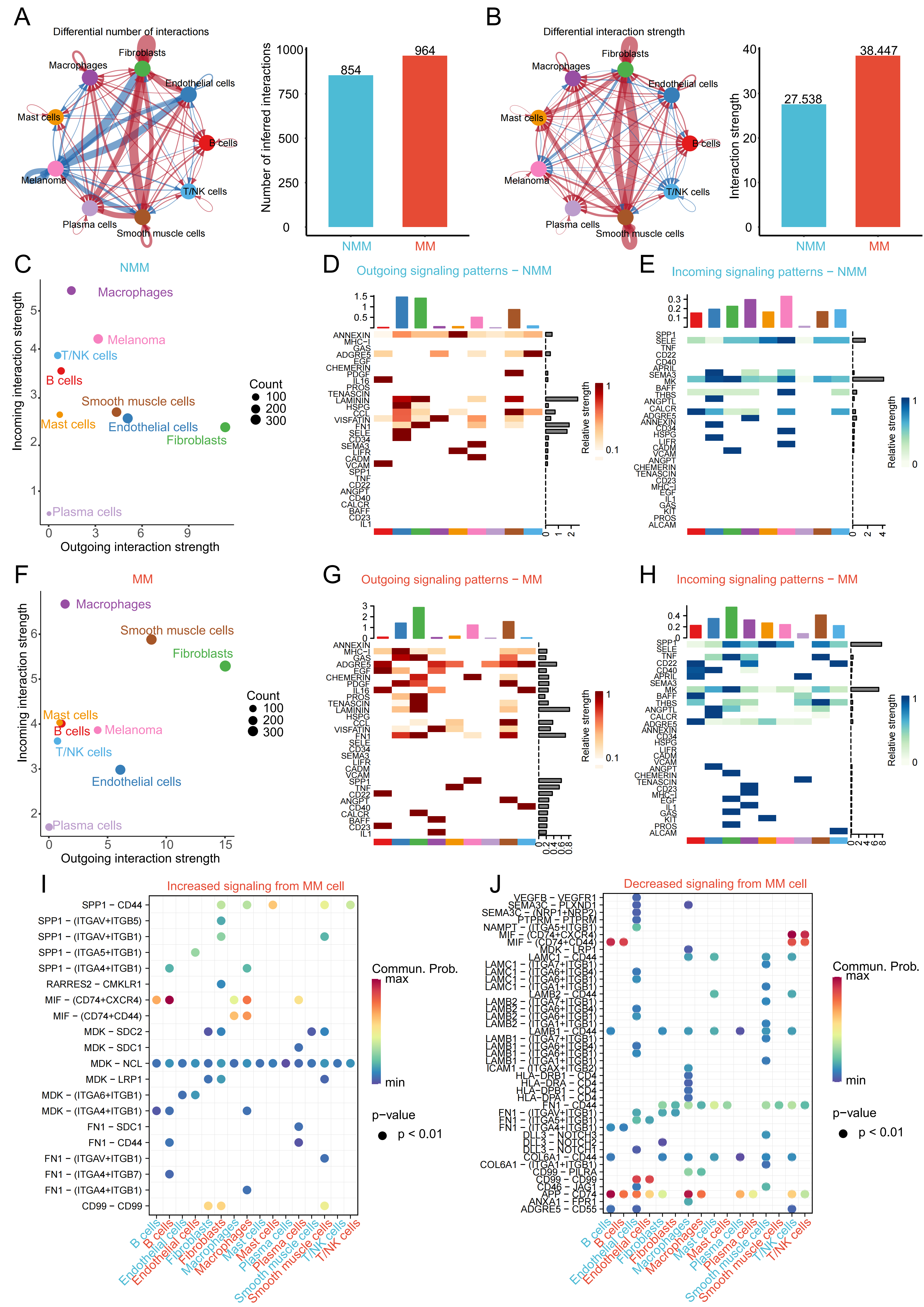

Supplement: Supplementary file 3 — Supplemental Material Figure 2 [file 41420_2023_1678_MOESM3_ESM.png]

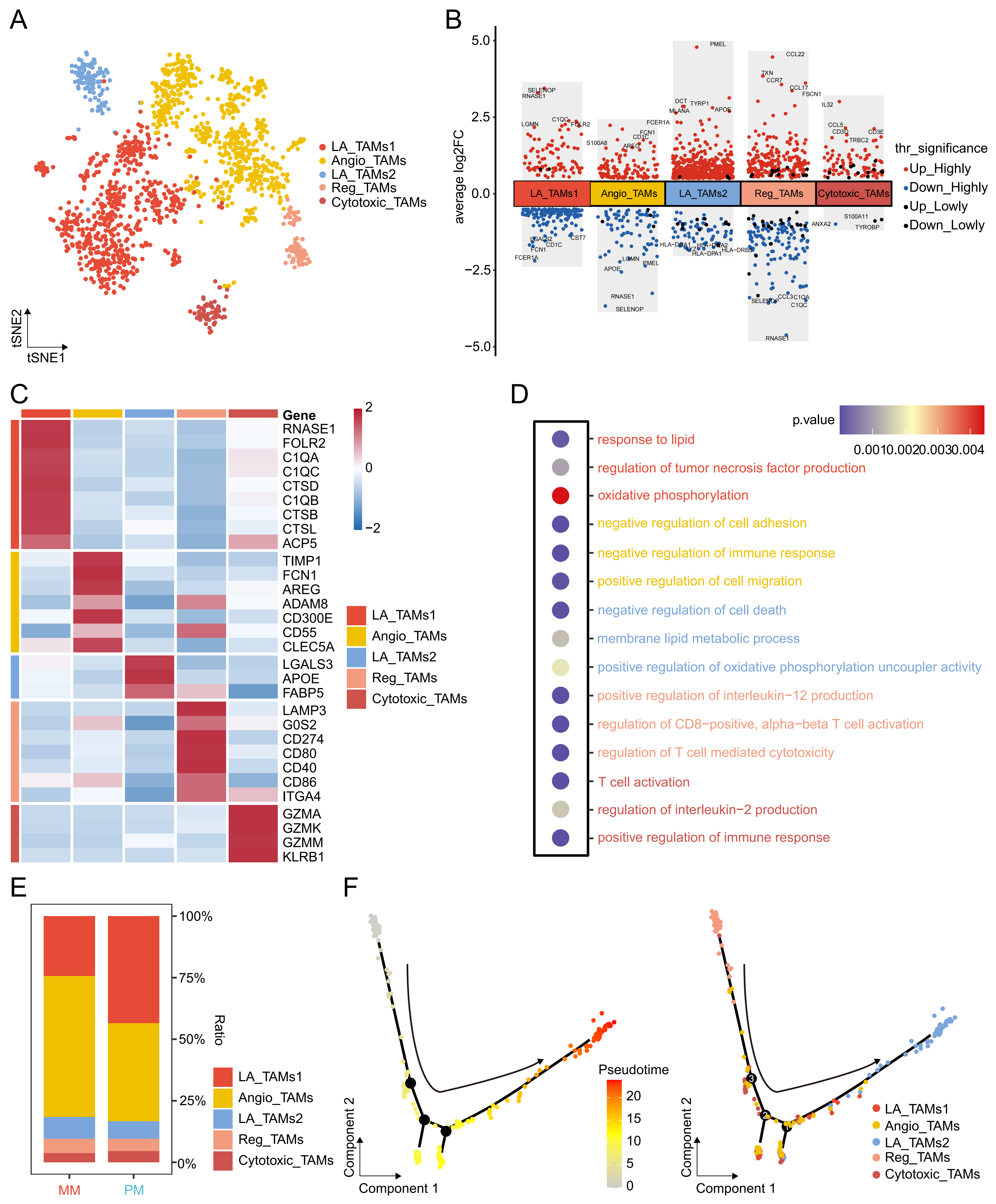

Supplement: Supplementary file 4 — Supplemental Material Figure 3 [file 41420_2023_1678_MOESM4_ESM.png]

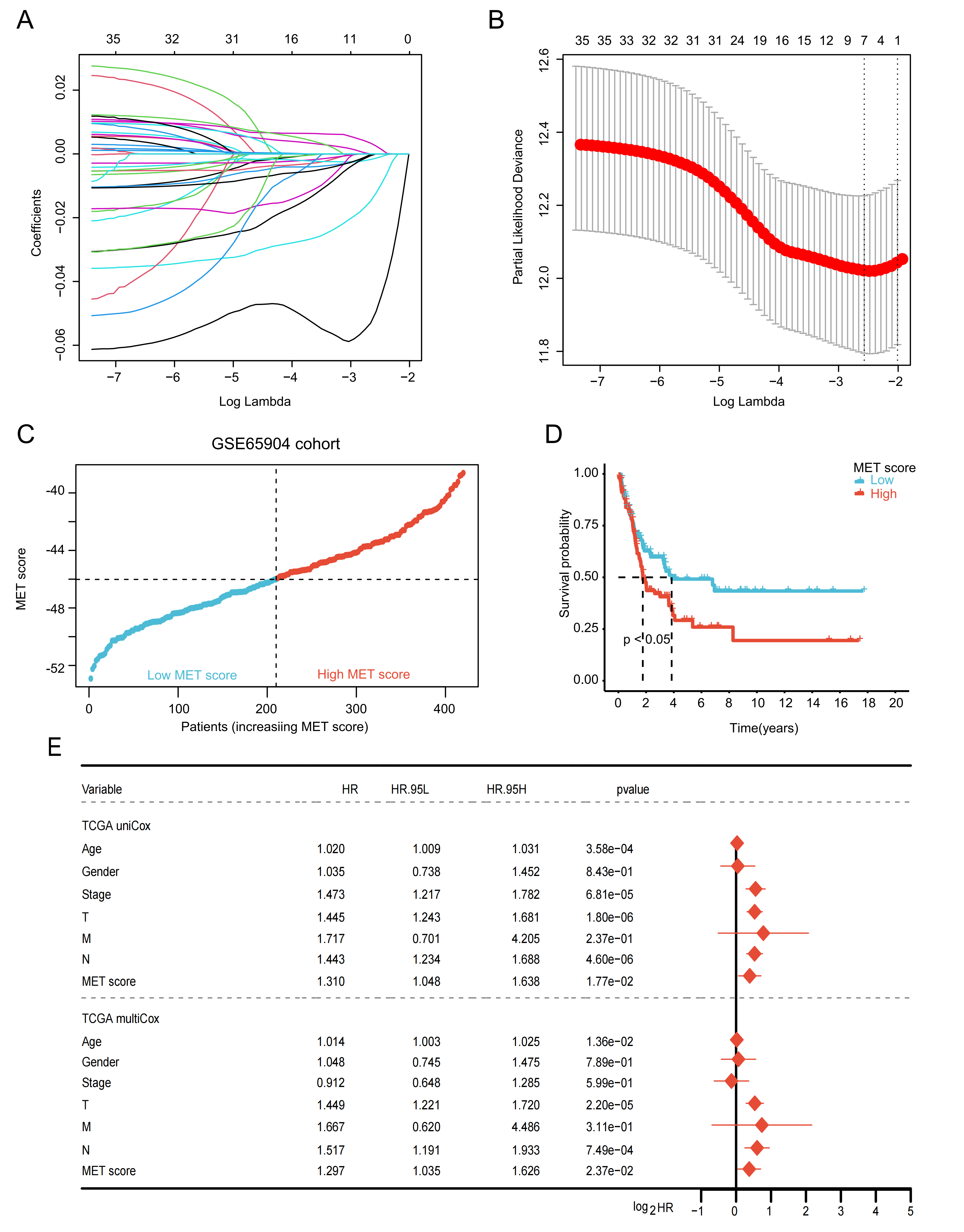

Supplement: Supplementary file 5 — Supplemental Material Figure 4 [file 41420_2023_1678_MOESM5_ESM.png]

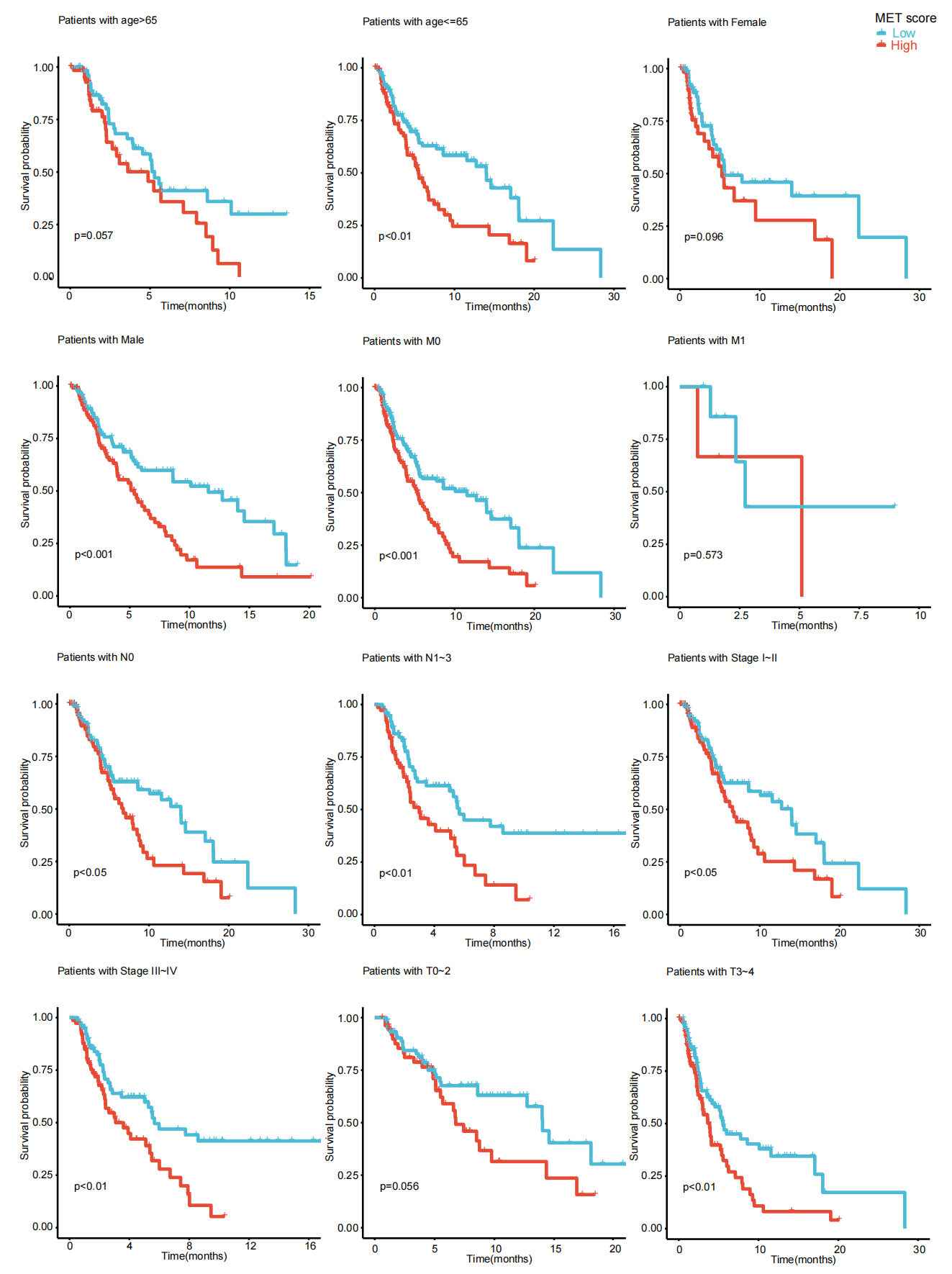

Supplement: Supplementary file 6 — Supplemental Material Figure 5 [file 41420_2023_1678_MOESM6_ESM.png]

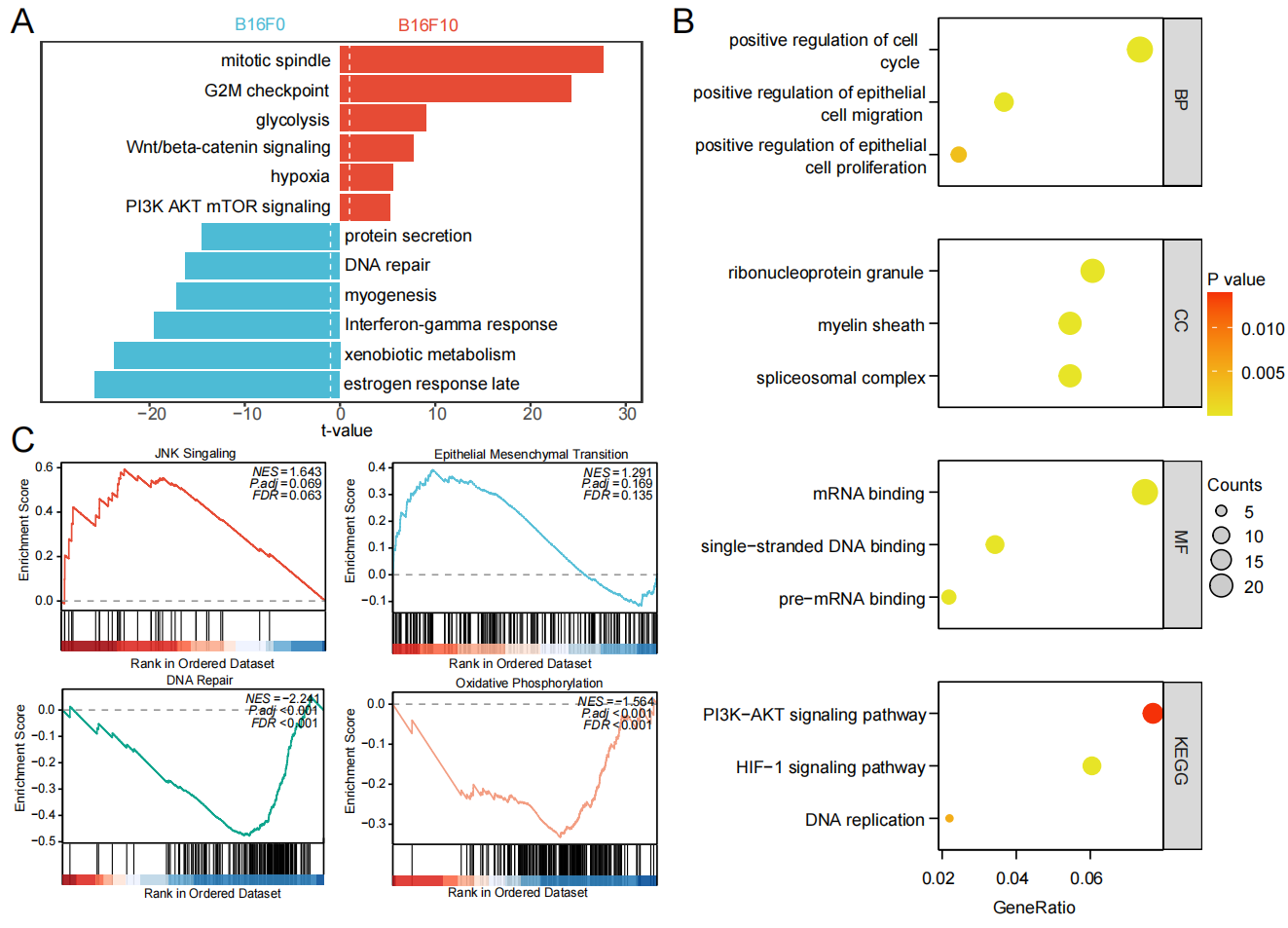

Supplement: Supplementary file 7 — Supplemental Material Figure 6 [file 41420_2023_1678_MOESM7_ESM.png]

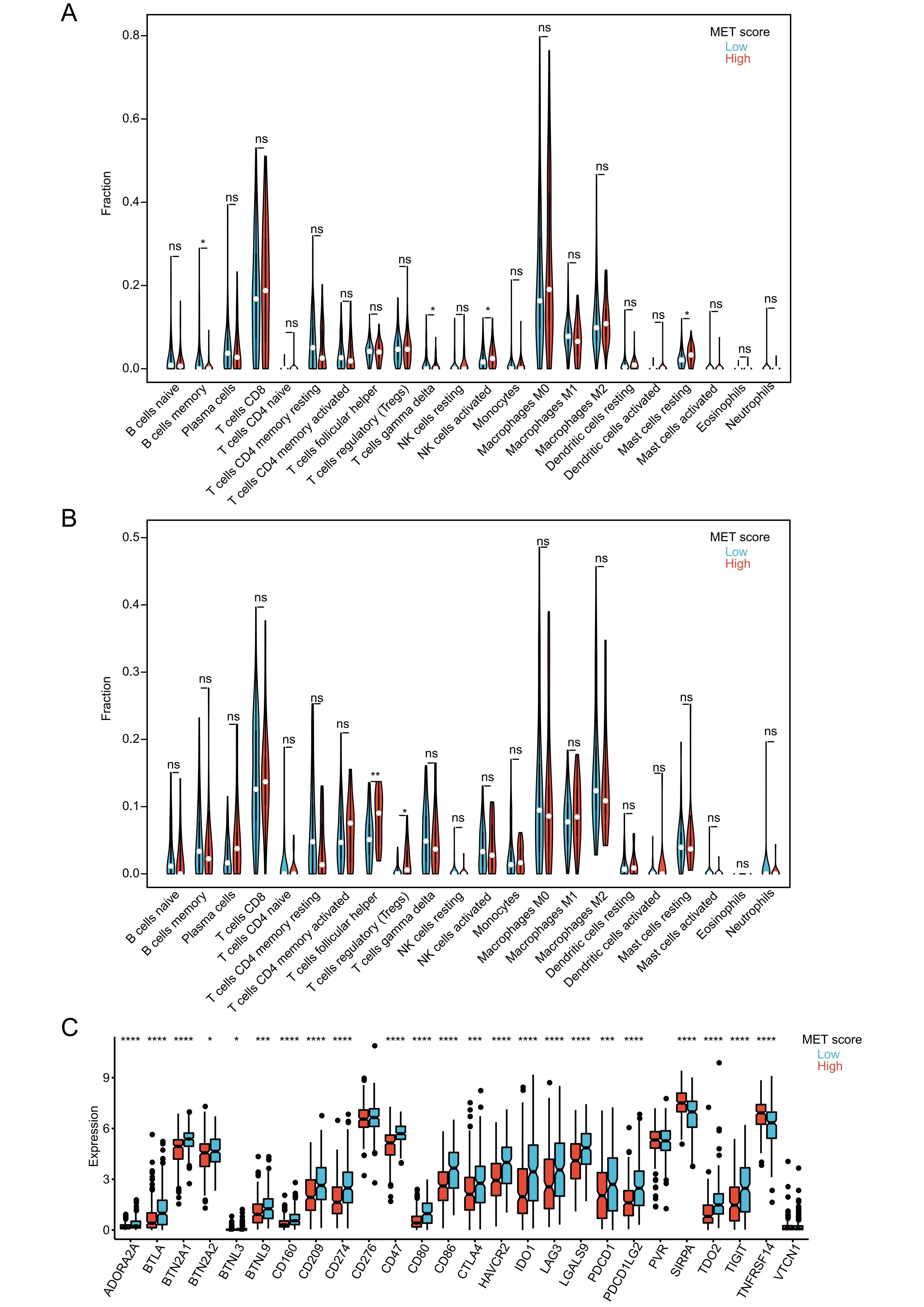

Supplement: Supplementary file 8 — Supplemental Material Figure 7 [file 41420_2023_1678_MOESM8_ESM.png]

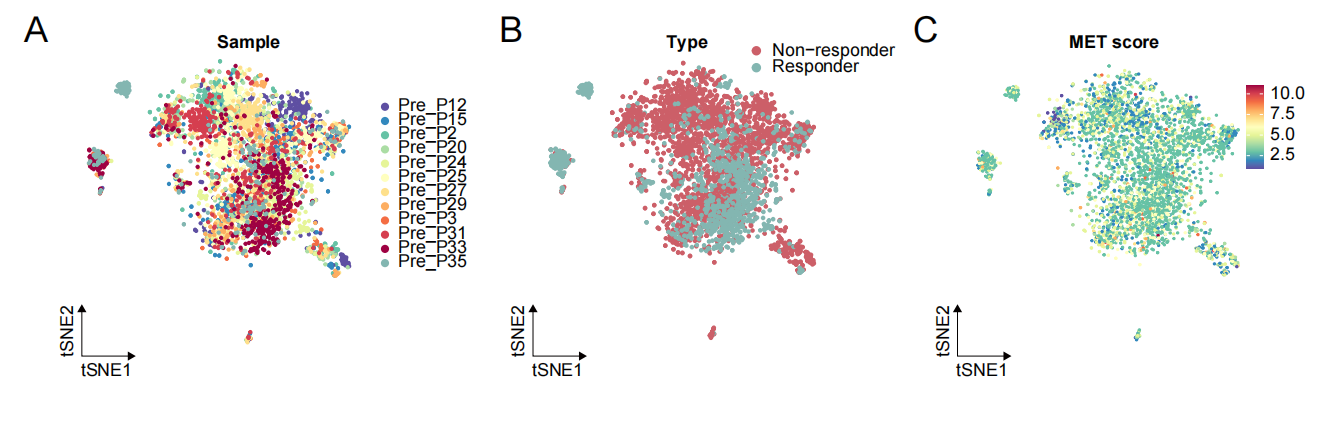

Supplement: Supplementary file 9 — Supplemental Material Figure 8 [file 41420_2023_1678_MOESM9_ESM.png]
